# Prevalence of triglyceride deposit cardiomyovasculopathy among patients with acute coronary syndrome

**DOI:** 10.1093/ehjacc/zuae005

**Published:** 2024-01-11

**Authors:** Yusuke Nakano, Ken-ichi Hirano, Tomohiro Onishi, Hirohiko Ando, Tetsuya Amano

**Affiliations:** Department of Cardiology, Aichi Medical University, 1-1 Yazakokarimata, Nagakute, Aichi 480-1195, Japan; Department of Triglyceride Science, Laboratory of Cardiovascular Disease, Novel, Non-invasive, and Nutritional Therapeutics, Graduate School of Medicine, Osaka University, Suita, Osaka, Japan; Department of Cardiology, Aichi Medical University, 1-1 Yazakokarimata, Nagakute, Aichi 480-1195, Japan; Department of Cardiology, Aichi Medical University, 1-1 Yazakokarimata, Nagakute, Aichi 480-1195, Japan; Department of Cardiology, Aichi Medical University, 1-1 Yazakokarimata, Nagakute, Aichi 480-1195, Japan

Triglyceride deposit cardiomyovasculopathy (TGCV) is an emerging cardiovascular disorder exhibiting diffuse narrowing of the coronary arteries due to defective intracellular lipolysis of triglyceride (TG). It is associated with a high risk of major cardiovascular events. However, many patients may remain undiagnosed due to limited awareness of the disease and the necessity for specific diagnostic tests that indicate TG accumulation in the myocardium.^1–4^ Given that coronary artery disease (CAD) is a leading cause of death worldwide, determining the frequency of TGCV and stratifying it as a risk factor for CAD is crucial. However, no real-world data exist regarding the prevalence of TGCV among patients with common cardiovascular diseases. Therefore, we conducted a retrospective study to evaluate the prevalence of TGCV among patients with common cardiovascular diseases presenting as acute coronary syndrome (ACS).

From May 2012 to July 2017, consecutive 400 patients underwent urgent coronary angiography for suspected ACS and 123I-β-methyl-p-iodophenyl pentadecanoic acid (BMIPP) scintigraphy for evaluation of intracellular TG lipolysis (Aichi Medical University, Nagakute, Japan) were retrospectively enrolled as the main population. From them, all patients with diabetes mellitus (DM) were selected as the sub-population. Among the populations, TGCV was diagnosed based on the latest diagnostic criteria, and we investigated the prevalence of TGCV among eligible patients, as detailed in the Supplementary material.

*Table 1* provides baseline patients’ clinical characteristics. In the main group (*n* = 400), 17 patients had definite TGCV. Among them, 12 were men with an average age of 71.0 years and an average body mass index (BMI) of 23.2 kg/m². All TGCV patients had diffuse narrowing coronary arteries, with a mean BMIPP washout rate (BMIPP-WR) of 5.4%. Because of not displaying typical Jordans’ anomaly, all diagnosed patients were classified as idiopathic type. In regard to baseline characteristics, significant differences were observed between patients with TGCV and those without TGCV, concerning diffuse narrowing coronary artery and the mean BMIPP-WR. *Figure 1A* illustrates the distribution of BMIPP-WR in the main group. Among these patients, 20 (5.0% of total) had a BMIPP-WR below 10.0%, a key diagnostic criterion for TGCV, with 17 (4.3% of total) having definite TGCV.

**Figure 1:**
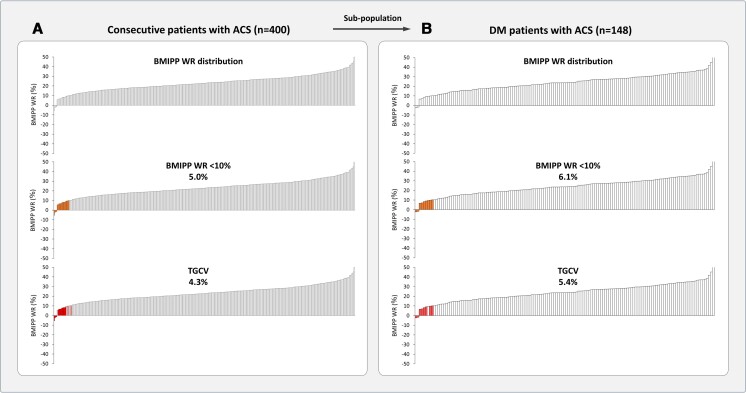
Distribution of BMIPP-WR and prevalence of TGCV. (*A*) The distribution of BMIPP-WR among 400 consecutive patients with ACS. Twenty of these patients (5.0%, coloured area) had BMIPP-WR < 10%, and 17 patients had definitive TGCV, accounting for 4.3% (coloured area) of total patients. (*B*) From the main population, 148 patients with diabetes mellitus were selected as the sub-population. Nine of these patients (6.1%, coloured area) had BMIPP-WR < 10%, and eight of them had definitive TGCV, accounting for 5.4% (coloured area). TGCV, triglyceride deposit cardiomyovasculopathy; BMIPP, 123I-β-methyl-p-iodophenyl pentadecanoic acid; WR, washout rate; ACS, acute coronary syndrome; DM, diabetes mellitus.

**Table 1:** Baseline clinical characteristics

	A. Consecutive patients with ACS (*n* = 400)				B. DM patients with ACS (*n* = 148)			
	All	TGCV	Non-TGCV	*P*-value	All	TGCV	Non-TGCV	*P*-value
No. of patients, *n*	400	17	383		148	8	140	
Men, *n* (%)	307 (76.8)	12 (70.6)	295 (77.0)	0.539	117 (79.1)	7 (87.5)	110 (78.6)	0.546
Age, y	66.6 ± 12.3	71.0 ± 10.7	66.4 ± 12.4	0.135	68.0 ± 11.2	72.5 ± 10.1	67.7 ± 11.2	0.241
BMI	23.8 ± 4.0	23.2 ± 4.3	23.9 ± 4.0	0.481	23.8 ± 3.5	23.2 ± 2.6	23.8 ± 3.6	0.256
STEMI, *n* (%)	206 (51.5)	8 (47.1)	198 (51.7)	0.708	49 (33.1)	2 (25.0)	47 (33.6)	0.616
Diffuse narrowing coronary artery, *n* (%)	71 (17.8)	17 (100.0)	54 (14.1)	<0.001	61 (41.2)	8 (100.0)	53 (37.9)	<0.001
BMIPP washout rate, %	23.2 ± 8.8	5.4 ± 4.9	24.0 ± 8.0	<0.001	24.1 ± 10.0	5.9 ± 5.0	25.1 ± 9.1	<0.001
Days from CAG to BMIPP scintigraphy, d (IQR)	9 (5, 13)	7 (−3, 11)	9 (5, 13)	0.313	9 (4, 19)	7 (4, 12)	9 (4,20)	0.650
Prior CABG, *n* (%)	46 (11.5)	3 (17.6)	43 (11.2)	0.417	34 (23.0)	3 (37.5)	31 (22.1)	0.315
Current smoker, *n* (%)	121 (30.3)	4 (23.5)	117 (30.5)	0.538	40 (27.0)	2 (25.0)	38 (27.1)	0.894
Hypertension, *n* (%)	253 (63.3)	9 (52.9)	244 (63.7)	0.368	81 (54.7)	4 (50.0)	77 (55.0)	0.782
Dyslipidaemia, *n* (%)	171 (42.8)	6 (35.3)	165 (43.1)	0.525	82 (55.4)	3 (37.5)	79 (56.4)	0.295
Diabetes, *n* (%)	148 (37.0)	8 (47.1)	140 (36.6)	0.380	148 (100.0)	8 (100.0)	140 (100.0)	1.000
HbA1c, %	6.5 ± 1.1	6.6 ± 1.2	6.5 ± 1.1	0.701	7.2 ± 1.4	7.4 ± 1.3	7.2 ± 1.4	0.755
Insulin, *n* (%)	19 (4.8)	2 (11.8)	17 (4.4)	0.165	19 (12.8)	2 (25.0)	17 (12.1)	0.290

ACS, acute coronary syndrome; TGCV, triglyceride deposit cardiomyovasculopathy; DM, diabetes mellitus; BMI, body mass index; STEMI, ST-elevation myocardial infarction; BMIPP, 123I-β-methyl-p-iodophenyl pentadecanoic acid; CAG, coronary angiography; IQR, interquartile range; CABG, coronary artery bypass grafting.

In the sub-group (*n* = 148), eight patients had definite TGCV. Seven were men, with an average age of 72.5 years and an average BMI of 23.2 kg/m². Their mean BMIPP-WR was 5.9%, with two receiving insulin treatment. Regarding patient characteristics, significant differences were observed between patients with TGCV and those without TGCV, in terms of diffuse coronary artery stenosis and the mean BMIPP-WR. *Figure 1B* shows BMIPP-WR distribution in the sub-group, with nine patients (6.1%) exhibiting a BMIPP-WR below 10.0% and eight of them (4.3%) having definite TGCV.

The present study specifically assessed the prevalence of TGCV among patients with ACS and patients with ACS and DM (4.3% and 5.4%, respectively).

Triglyceride deposit cardiomyovasculopathy is a newly identified cardiovascular disorder, and most of them have developed CAD with diffuse narrowing,^1,3–5^ some of which underwent several rounds of percutaneous coronary intervention for stent restenosis.^3^ In Japan, currently, ∼ 600 patients, including patients in the present study have been diagnosed with definitive TGCV. However, the prevalence of TGCV in clinical settings for acute coronary care is unknown. By investigating high-risk patient cohorts, such as ACS and ACS with DM, we aimed to enhance the understanding of the frequency and impact of TGCV on mortality due to cardiovascular causes worldwide. We have reported cases of TGCV with atherosclerosis regression following supplemental tricaprin/trisdecanoin, a medium-chain TG, which might be a potential therapeutic option in patients with TGCV.^5^

We acknowledge the limitations with a small sample, including only Japanese patients. Moreover, this retrospective cohort study might underestimate the prevalence of TGCV to some, but likely minimal, degrees, because we noted a paradoxical and transient increase of BMIPP-WR in 5 patients with TGCV related to coronary events among 600 patients ever identified; even its detailed mechanism(s) remain unknown.^4^ Clinical studies with large cohorts are required to corroborate the results of the present study.

The analysis revealed the prevalence of TGCV among populations with ACS. It is important to consider TGCV for risk stratification in these patients.

## Supplementary material

Supplementary material is available at *European Heart Journal: Acute Cardiovascular Care* online.

## Supplementary Material

zuae005_Supplementary_Data

## Data Availability

The data underlying this article will be shared on reasonable request to the corresponding author.
