# Supplementary material for: Prevalence of triglyceride deposit cardiomyovasculopathy among patients with acute coronary syndrome
Source: Eur Heart J Acute Cardiovasc Care. 2024 Jan 11;13(3):293–5. doi: 10.1093/ehjacc/zuae005 (PMC10927025; doi:10.1093/ehjacc/zuae005)
Supplement: zuae005_Supplementary_Data [file zuae005_supplementary_data.docx]

**Supplemental material**

**Description of the diagnostic criteria for ACS and DM**

ACS diagnosis is based on 2023 ESC Guidelines for the management of acute coronary syndromes. [European Heart Journal. 2023, <https://doi.org/10.1093/eurheartj/ehad191>]

DM diagnosis is based on the diagnostic criteria of DM by the Japan Diabetes Society. [Diabetology International. 2010;1(1):2 20]

**Description of the diagnostic criteria for TGCV**

According to these criteria [Ann Nucl Cardiol 2020; 6(1):99-104], at least one essential item and one major item are required for a definitive diagnosis. The essential items reflecting defects in intracellular lipolysis include the following: 1) decreased BMIPP WR on myocardial scintigraphy (<10%), 2) presence of lipid droplets in myocardial cells, and 3) accumulation of myocardial triglycerides, as determined using magnetic resonance spectroscopy/computed tomography. The major items indicating clinical severity are as follows: 1) reduced left ventricular ejection fraction (<40%), 2) diffuse narrowing coronary arteries, and 3) typical Jordans' anomaly in peripheral leukocytes [Acta Med Scand. 1953;145(6):419 423]. TGCV is classified into primary and idiopathic TGCV, whether PNPLA2 mutations exist or not, respectively. Primary TGCV usually presents with typical Jordans' anomaly.

**Ethics**

The present study was conducted according to the principles outlined in the Declaration of Helsinki. The study protocol was approved by the Ethics Committee of Aichi Medical University, and written informed consent was obtained from all patients or their relatives.

**Statistical analysis**

Continuous variables with a normal distribution in patients backgrounds are expressed as mean ± standard deviation and median (interquartile range), and comparisons between the 2 groups were appropriately performed using unpaired Student’s *t* test and Mann-Whitney U test. Categorical variables are presented by patient number (%), and were analyzed using Chi-squared test. All statistical analyses were performed using IBM SPSS Statistics for Windows, version 25 (IBM Corp., Armonk, NY, USA).
